# Medical Department of the University of Buffalo

**Published:** 1858-04

**Authors:** 


### EDITORIAL DEPARTMENT.

*The Medical Department of the University of Buffalo.*—Twelve years ago, when the Med. department of the University of Buffalo was organized the condition of the medical profession and of medical colleges, was very different from the present state of things. Then, the office of every medical practitioner contained one or more students, and those who had attained to any degree of eminence, were absolutely flooded with applications. In a recent conversation with some of the original teachers in the Buffalo school, who were of course close observers of the state of the colleges at that time, our attention was called to the fact which we have just stated, and the immense contrast which is now presented. The Buffalo Medical College commenced with nearly eighty pupils, and that, after a notice of only three months. In the session of 1849–50, the class numbered 115, which, we believe, was the largest class ever assembled at any season. As far as our recollection serves, it was about that time that the class at the Louisville University, numbering upwards of 400, was almost too large for the capacious halls of their noble building. The colossal Jefferson then merited the title, as much and even more than it does now, when it can boast that it assembles the largest medical class in the world. All around us the schools were full, the professors paid, and the medical men not connected with any institution croaking about the crowding of the profession, low standard of merit, *et cetera.* But now the contrast will be striking and apparent to every one. We have merely mentioned particularly one or two of the medical schools, but it is well known that at the time to which we refer, all were highly prosperous; and in looking now for some of them, we find that they have absolutely given up the ghost; some are dragging out a miserable existence with nominal fees, weakened faculties, and almost no students; and others, by an entire abolition of fees, are preying upon the vitals of their wobegone neighbors, contributing as much as possible to lower the professional standard, and reduce their competitors the alternative of extinction, or the level to which *they* have fallen. In spite of all this, however, the Philadelphia and New York schools hold their own, making now, with the exception of the Nashville school, the only exception to the general rule.

The Buffalo school has not suffered in this respect out of proportion to the general diminution of medical students throughout the land. In one thing, however, it is an exception, its faculty is strong, and its dignity has not suffered from the reduction of fees; added to this, are a determination and a power to weather the storm through, and clinical advantages which are not surpassed even in Philadelphia or New York. We feel attached to the Buffalo Medical College, from the high standard of honor with which it commenced, and which it has invariably sustained, and our readers especially will sympathize with our feelings of gratitude for the valuable aid which the Journal has always received from the faculty, and which is one of the causes which has been most instrumental in placing it in the position which we are proud to say it occupies.

The strength of the faculty, which will compare favorably with any in the country; the clinical opportunities, the Hospital of the Sisters of Charity being situated directly behind the college building, and students having opportunities of observing medical and surgical cases at the bed side, under the guidance of the ablest clinical teachers in the country; the maternity hospital, also situated within a stone's throw of the college, which is under the direction of of the Professor of Obstetrics, and to which all the graduating class have repeated access: are advantages to which we hope and are confident that none of our readers will be insensible, when recommending to their students a place for the prosecution of their winters tudies. All these are certain to make the school ultimately in the highest degree successful, in point of numbers, and we are as convinced of its inevitability as though we already saw 200 students in its halls.

We regret to state that Prof. Hunt, our senior editor, has announced his intention of resigning his chair. This is owing to engagements which he has formed with our most prominent daily paper, and which closely occupy his time. We copy resolutions passed by the faculty expressive of their regret at the step which he proposes to take. The lectures on that branch, will be given this year by Dr. A. W. Nichols, formerly Demonstrator of Anatomy, who has been appointed lecturer, and who is already favorably known to our readers as reporter of cases occurring in the wards of Prof. Flint, at the hospital, and by other papers of merit which have appeared from time to time in this Journal.

"Professor Sanford B. Hunt having announced his intention of resigning the chair of *General and Descriptive Anatomy and Physiology* in the University of Buffalo, the medical faculty of the University, desirous of giving expression to the sentiments occasioned by this announcement, adopt unanimously the following resolutions:

"*Resolved*, That the faculty receive this announcement with profound regret, feeling that by the resignation of Prof. Hunt, they lose a colleague whose services as a professor have been most valuable to the college, and with whom as a friend and associate their relations have ever been a source of pleasure and advantage.

"*Resolved*, That in tendering to Prof. Hunt their warmest wishes for his success in those labors which have deprived them of a valuable colleague, gratified by the assurance that his interest in the science and profession of medicine will remain unabated, and that his exertions will continue in behalf of medical literature, which is already so much indebted to his talents, scientific attainments and manly independence.

"*Resolved*, That a copy of these resolutions be transmitted to Prof. Hunt by the Registrar of the Faculty, and that copies be furnished for publication in the Buffalo Medical Journal, and in the daily papers of this city.

"GEOREG HADLEY, Registrar.

"Buffalo, Feb. 24, 1858."

We also copy from the Commercial Advertiser, a report of the annual commencement, by the senior editor. At the meeting of the Council of the University of Buffalo, held on the 24th ult., Henry W. Rogers, Esq., was elected member of the council, to fill the vacancy occasioned by the death of the late Dr. Thomas M. Foote.

The following medical gentlemen were added to the list of curators of the medical department of the University:

| | |
|---|---|
| Charles H. Wilcox, M. D., . . | Buffalo. |
| Lewis P. Dayton, M. D., . . . | " |
| W. McCullum, M. D., . . . | Lockport. |
| W. B. Gould, M. D., . . . . | " |
| T. H. Blake, M. D., . . . . | Dansville. |
| B. L. Hovey, M. D., . . . . | " |
| J. Beatie, M. D., . . . . | Geneva. |
| C. E. Washburn, M. D., . . . | Fredonia. |
| T. D. Strong, M. D., . . . . | Westfield. |
| D. D. Robinson, M. D., . . . | Hornellsville. |

The annual commencement of the University of Buffalo, medical department, took place at American Hall, Feb. 23d, in the presence of an audience more than usually large for such occasions, and comprising a large modicum of the *élite* of the city. Among those present on the platform were ex President Fillmore, in his capacity of Chancellor; Hon. Geo. R. Babcock, and other members of the Council; the Faculty of the school, among them Prof. Mack, of St. Catherines, C. W., a new face to Buffalonians; and a considerable number of Curators from abroad who were here in pursuance of their duty as examiners. We saw among them Drs. Briggs and Whitbeck, of Rochester, Dr. Cotes, of Batavia, and Dr. Charles, of Angelica.

The services were opened by an impressive and appropriate prayer by Rev. Dr. Burtis, in which he feelingly alluded to the loss sustained by the school in the death of Dr. Foote, who has been a member of its Council from the beginning.

Prof. Rochester, Dean of the Faculty, then announced that the distinction of special mention had been conferred on five gentlemen, whose theses were especially meritorious, and that the honor of an authority to publish had been conferred on two of these, viz: Bernard Monahan, of Buffalo, and Alonzo W. Gilbert, of Bowmansville, C. W.

The degrees were then conferred by Chancellor Fillmore, in the usual form, on the following young gentlemen:

| | |
|---|---|
| Wm. Henry Butler, | Buffalo, N. Y. |
| Walter Butler Coventry, | Utica, N. Y. |
| Thomas Cushing, | North East, Pa. |
| Alonzo W. Gilbert, | Bowmanville, C. W. |
| Wilmot Hall Garlick, | Cleveland, Ohio. |
| George W. Jones, | Belleville, C. W. |
| Augustus F. D. Jansen, | Buffalo, N. Y. |
| Bernard Monahan, | Buffalo, N. Y. |
| Joseph Tripp, | Morenci, Mich. |

The charge to the graduates was given by Prof. Moore, of Rochester. It was a thoughtful and careful statement of the essentials of the true physician, involving a discussion of the claims of medicine to the title of an actual science, an honest and fearless statement of what is known and what is unknown, and an earnest protest against a too confident trust in the efficacy of drugs. Dr. Moore asserted that diagnosis was the first element in the character of the true physician; that the capacity to interpret the meaning of symptoms, and to predicate from them the actual phenomena which were occurring concealed within the system, was much more valuable *to the patient* than the extensive knowledge of druggists, formulæ and recipes. *To the physician,* however, to his pocket and fame, drugs are the amulet which is indispensable. The physician who gives advice is, by the masses, rejected for him who gives physic. The people have no appreciation of the silent forces of nature, and how gently and easily they may be guided by the sklll of the acute, investigating physician. They wish physic, pills, boluses and potions, big or little, and as long as the appetite exists they will have them.

We could occupy much space pleasantly in a sketch of Prof. Moore's admirable charge, but should fail to do justice to its broad and philosophic spirit, its catholicity, and its high merit as a literary production.

The address thus completed, the benediction was given by Rev. Dr. Burtis, and the audience broke up. The young men thus started out upon the career of an arduous and responsible profession, are, it is believed, of such scholarship and talent as to do honor to themselves in its ranks.

